# New Volleyballenes: Y_20_C_60_ and La_20_C_60_

**DOI:** 10.1038/srep30875

**Published:** 2016-08-04

**Authors:** Jing Wang, Ying Liu

**Affiliations:** 1 Department of Physics and Hebei Advanced Thin Film Laboratory, Hebei Normal University, Shijiazhuang 050024, China; 2 National Key Laboratory for Materials Simulation and Design, Beijing 100083, China

## Abstract

Two new stable Volleyballenes, the Y_20_C_60_ and La_20_C_60_ molecular clusters, are proposed on the basis of first-principles density functional theory. In conjunction with recent findings for the scandium system, these findings establish Volleyballene *M*_20_C_60_ molecules as a general class of stable molecules within the fullerene family. Both Y_20_C_60_ and La_20_C_60_ molecules have *T*_*h*_ point group symmetries and relatively large HOMO-LUMO gaps.

Since the first observation of the C_60_ fullerene molecule^1^,^2^, much effort has been invested in the study of this novel molecular cluster. In the fullerenes, all the atoms are C atoms and they form a hollow sphere comprised of pentagonal and hexagonal rings. Very recently, on the basis of density functional theory (DFT) calculations, an exceptionally stable hollow cage, composed of 20 Sc atoms and 60 C atoms, the Volleyballene Sc_20_C_60_, was reported^3^. This molecular cluster has a *T*_*h*_ point group symmetry and a volleyball-like shape. This Volleyballene was the first buckyball to be spiked with metal atoms and is awaiting synthesis^4^,^5^,^6^,^7^,^8^.

If the Volleyballene Sc_20_C_60_ does actually exist, it is expected that other early transition metals should be capable of forming molecules of a similar type, and might also display unusual stability. We have therefore extended our work to other transition metal systems, with particular attention to elements with a single d electron: yttrium (Y) and lanthanum (La). As in the case of Sc_20_C_60_, the *M*_20_C_60_ (*M* = Y and La) molecules also are found to display an enhanced stability with the volleyball-like shape. In the following, the stability and electronic properties of the Volleyballenes Y_20_C_60_ and La_20_C_60_ are investigated through their bonding characters and the vibrational frequencies, as well as through molecular dynamics simulations.

Figure 1 shows the configurations of the two new Volleyballenes *M*_20_C_60_ (*M* = Y and La), which both have *T*_*h*_ point group symmetries within a tolerance of 0.1 Å. Similar to the case of Sc_20_C_60_, the new Volleyballenes are composed of six *M*_8_C_10_ subunits arranged in a crisscross pattern. In each *M*_8_C_10_ subunit, 10 carbon atoms form two head-to-head connected carbon pentagons (C-pentagon), and 8 transition-metal atoms form a single transition-metal octagon (*M*-octagon). The two connected C-pentagons are surrounded by the *M*-octagon, to give a structure that resembles the panels of a volleyball.

The 20 transition metal atoms link to form 12 suture lines with the average distances between transition-metal atoms being 3.411 Å for Y-Y and 3.617 Å for La-La. For the C-pentagons of the Y_20_C_60_ molecule, the lengths of the C-C bonds lie in the range 1.449–1.460 Å. Along with a 1.485 Å C-C bond connecting the two C-pentagons, the average C-C bond length is found to be 1.455 Å. The average Y-C bond length is 2.396 Å.

For La_20_C_60_, the C-C bond lengths are in the range 1.450–1.456 Å and the C-C bond connecting the two C-pentagons has a length of 1.490 Å, resulting in an average C-C bond length of 1.457 Å. The La-C bond length is 2.565 Å. Both the average C-C and *M*-C bond lengths, as well as the average *M*-*M* distance, in La_20_C_60_ are larger than the corresponding distances in Y_20_C_60_, indicating a larger-sized cage for La_20_C_60_. The reason may lie with the relatively larger atomic radius of La. All the calculated data including the binding energies per atom, are listed in Table 1. For the new Volleyballenes, the binding energies per atom are 6.622 and 6.565 eV, for Y_20_C_60_ and La_20_C_60_, respectively. For more details see Section I of the Supplementary Information.

The bonding characters of the Volleyballene *M*_20_C_60_ (*M* = Y and La) molecules were investigated by analyzing their deformation electron densities. The Volleyballenes Y_20_C_60_ and La_20_C_60_ have similar bonding characteristics, mainly due to their similar electron configurations, 4*d*^1^5*s*^2^ for the Y atom and 5*d*^1^6*s*^2^ for the La atom. On the whole, there is electron transfer from the transition metal atoms to the C atoms. Mülliken population analysis showed an average charge transfer of 0.95*e* from each Y atom to the neighboring C atoms for Y_20_C_60_, while for La_20_C_60_, the average charge transfer is 0.69 *e*. To better understand the chemical bonding, natural bonding orbital (NBO)^9^ analysis was employed, and it was found that the results of the natural population analysis (NPA) are in accord with those of Mülliken population analysis (Table 1). The NPA showed an average charge transfer of 0.92 *e* from each Y to the neighboring C atoms in Y_20_C_60_, while the charge transfer for La_20_C_60_ was 0.78 *e*. For the C atoms, there are obvious characteristics of *sp*^2^-like hybridization, and each C atom has three *σ* bonds. As with the Sc atoms in the Volleyballene Sc_20_C_60_, there are four lobes for each *M* atom in Volleyballene *M*_20_C_60_ (*M* = Y and La) molecules, pointing to the four neighboring C atoms. This strengthens the link between the *M*_8_C_10_ subunits.

The stability of the *M*_20_C_60_ molecules was further checked using ab initio molecular dynamics (MD) simulations with the constant-energy, constant-volume (NVE) ensemble. The simulation time step was set to be 1.0 *fs* with a total of 10000 dynamics steps. With initial temperatures of 2200 and 1800 K (~1100 and ~900 K effective temperatures) for Y_20_C_60_ and La_20_C_60_, respectively, the structures were not disrupted over the course of a 10.0 *ps* total simulation time. For more details see Section II of the Supplementary Information. Vibrational frequency analysis was also carried out, and no imaginary frequencies were found for either of the two Volleyballenes (Y_20_C_60_ and La_20_C_60_). These results indicate that the two new Volleyballenes, both have good kinetic and thermodynamic stabilities. Figure 2 shows the calculated Raman spectrums of Y_20_C_60_ and La_20_C_60_. The temperature was taken to be 300 K, and incident light of wavelength 488.0 nm was chosen in order to simulate a realistic Raman spectrum that can be compared to experimental results. The specific vibrational modes corresponding to the peaks of Raman spectrum are given in Section III of the Supplementary Information.

We then calculated the partial densities of states (PDOS) and the frontier molecular orbitals, including the highest occupied molecular orbital (HOMO) and the lowest unoccupied molecular orbital (LUMO) as shown in Fig. 3. From the contours of the HOMO orbitals, it can be seen that the HOMO orbitals are mostly localized on the C atoms. There is also obvious hybridization between C *p* and *M d* orbitals. For the LUMO, the orbital wave functions are mostly localized on the transition metal atoms, and have obvious *d* orbital characteristics. In one *M*_8_C_10_ subunit, four transition metals have 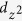-like orbital characteristics, and each of the other four has four pear-shaped lobes. The centers of all four lobes lie in one plane, which is perpendicular to the plane of the *M*_8_C_10_ subunit thus playing the role of a connection between *M*_8_C_10_ subunits. There is *sp*-*d* hybridization for the LUMO orbital. Noted that the LUMO of La_20_C_60_ is slightly different from that of Y_20_C_60_ at the same isosurface (0.015 e/Å^3^). For La_20_C_60_, the 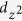-like orbital has obvious hybridization characteristics, with one pear-shaped region above the torus being larger than the one below the torus, while for Y_20_C_60_ this situation is not obvious. This may be due to La having a larger atomic radius than that of the transition metal Y. Close examination of the PDOS further confirms the hybridization characteristics of the HOMO and LUMO orbitals. All these results are consistent in demonstrating that hybridization between the *M d* orbitals and C *s*-*p* orbitals is essential for stabilizing the cage structure of *M*_20_C_60_ (*M* = Y and La).

For the Volleyballenes Y_20_C_60_ and La_20_C_60_, relatively large HOMO-LUMO gaps were found, as listed in Table 1. The HOMO-LUMO gaps are 1.395 eV for Y_20_C_60_ and 1.254 eV for La_20_C_60_. The large gaps are due mainly to the energies of the *d* atomic orbitals being much lower than those of the *p* orbitals. With relatively large HOMO-LUMO gaps, the two new Volleyballenes Y_20_C_60_ and La_20_C_60_ should be stable fullerene variants with moderately high chemical stability.

In summary, first-principles studies have identified two new stable Volleyballenes, Y_20_C_60_ and La_20_C_60_. In an initial report on the stability of Sc_20_C_60_^3^, we speculated that Sc_20_C_60_ might comprise one member of a Volleyballene family, and that other transition or rare-earth metals could also form stable *M*_20_C_60_ molecular clusters. This speculation now appears to have been borne out.

## Methods

The calculations were carried out with the exchange-correlation potential described by the Perdew-Burke-Ernzerhof (PBE) version of the general gradient approximation (GGA)^10^. The double-numerical basis plus polarized functions (DNP)^11^ was chosen. For the transition metal atoms, relativistic effects in the core were included using the DFT semi-core pseudopotentials (DSPP)^12^. All structures were fully relaxed, and geometric optimizations were performed with unrestricted spin and without any symmetry constraints as implemented in the DMol^3^ package^13^.

## Additional Information

**How to cite this article**: Wang, J. and Liu, Y. New Volleyballenes: Y_20_C_60_ and La_20_C_60_. *Sci. Rep.* **6**, 30875; doi: 10.1038/srep30875 (2016).

## Supplementary Material

Supplementary Information

## Figures and Tables

**Figure 1:**
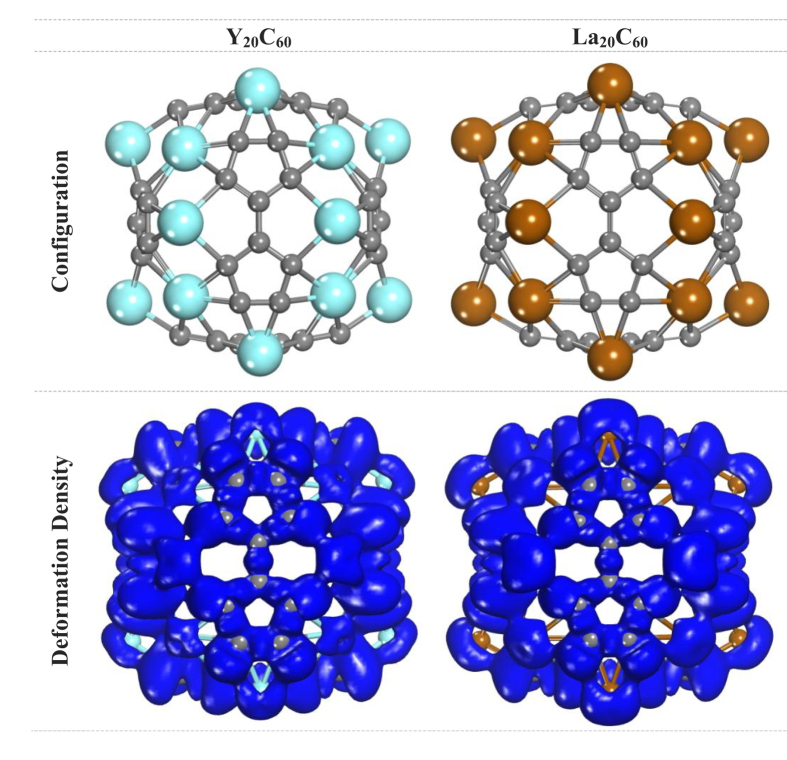
The configurations and deformation electron densities of Y_20_C_60_ and La_20_C_60_. The isosurface is taken to be 0.01 e/Å^3^.

**Figure 2:**
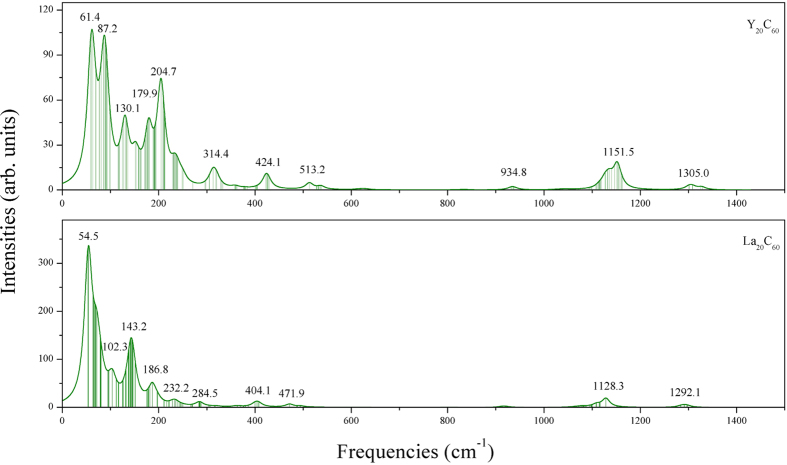
Simulated Raman spectrums for the Volleyballenes *M*_20_C_60_ (*M* = Y and La) at a temperature of 300 K and using 488.0 nm incident light. The Lorentzian smearing was set to be 20.0 *cm*^−1^. The labels show the frequencies corresponding to the peaks of the intensities.

**Figure 3:**
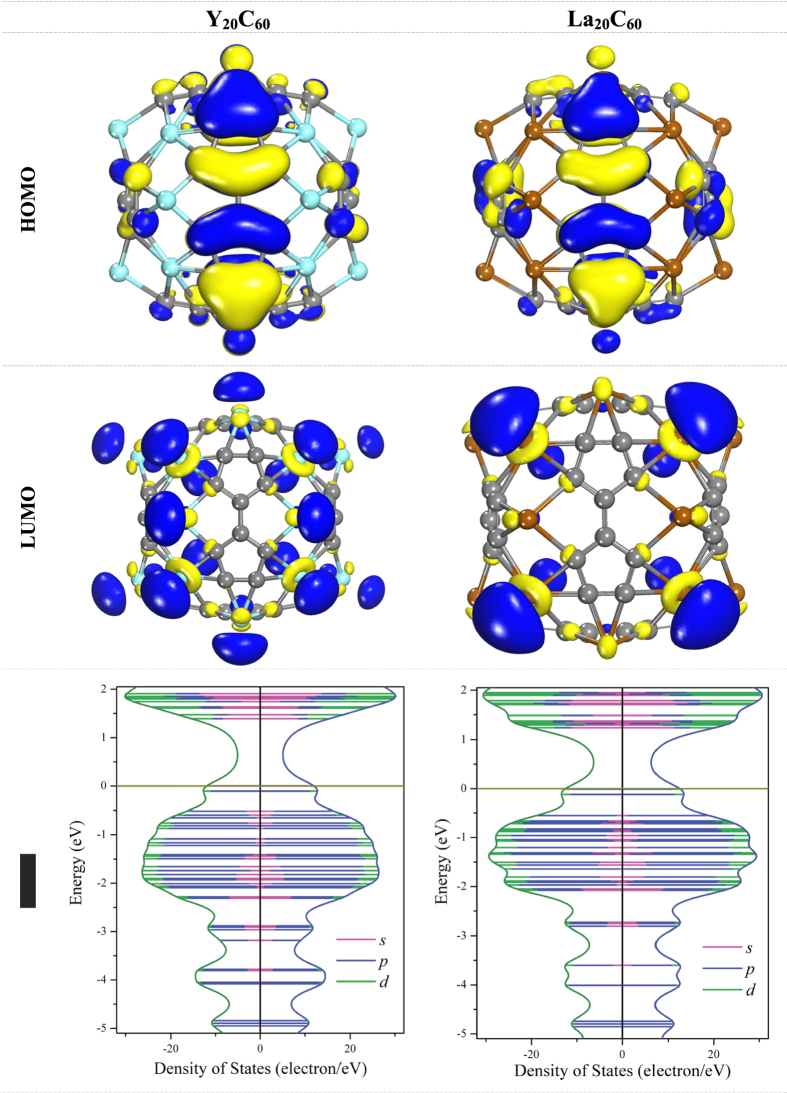
The HOMO and LUMO orbitals, and PDOS for *M*_20_C_60_ (*M* = Y and La). The isosurface for the orbitals is set at 0.015 e/Å^3^.

**Table 1:** Summary of the calculated results for *M*_20_C_60_ (*M* = Y and La).

	Sym.	*d*_1_	*d*_2_	*Q*_*M*_	*Q*_*NPA*_	*E*_*b*_	*E*_*g*_
Y_20_C_60_	*T*_*h*_	1.455	2.396	0.953	0.921	6.622	1.395
La_20_C_60_	*T*_*h*_	1.457	2.565	0.693	0.779	6.565	1.254

The data include the symmetry group (Sym.), the average C-C (*d*_1_) and *M*-C (*d*_2_) bond lengths, the average charge transfer from *M* to carbon atoms (*Q*_*M*_ for Mülliken analysis and *Q*_*NPA*_ for NBO analysis), the binding energy per atom (*E*_*b*_), and the HOMO-LUMO energy gap (*E*_*g*_) in units of Å for the lengths and eV for energy.
