# Supplementary material for: New Volleyballenes: Y20C60 and La20C60
Source: Sci Rep. 2016 Aug 4;6:30875. doi: 10.1038/srep30875 (PMC4973237; doi:10.1038/srep30875)
Supplement: Supplementary Information [file srep30875-s1.doc]

New Volleyballenes: Y_20_C_60_ and La_20_C_60_

Jing Wang^1^, and Ying Liu^1,2^,*

^1^Department of Physics and Hebei Advanced Thin Film Laboratory, Hebei Normal University, Shijiazhuang 050024, Hebei, China

^2^National Key Laboratory for Materials Simulation and Design, Beijing 100083, China

**Section I. Binding energies of Volleyballenes and Met-Cars**

In this section, we describe the calculation of the binding energies per atom for the *Volleyballenes* Y_20_C_60_ and La_20_C_60_, as well as for the Met-Cars Y_8_C_12_ and La_8_C_12_. Energy minimization was imposed on the Met-Car clusters with *D*_2_*_d_*, *C*_3_*_v_*, *D*_3_*_d_*, and *T_h_* symmetries, and the binding energies per atom were then calculated. Of the Met-Cars obtained, the *D*_3_*_d_*-based structures had the largest binding energy per atom, 6.046 eV for Y_8_C_12_ and 6.024 eV for La_8_C_12_. All the calculated results are listed in Table S1. From the data of Table S1, it can be seen that the binding energies per atom of the *Volleyballenes* are higher than those of the four corresponding Met-Cars, and the differences are in the range 0.576~1.123 eV for the Y systems and 0.541~0.991 eV for La systems.

| **Table S1.** The binding energies per atom (*E_b_*) of the Volleyballenes Y_20_C_60_ and La_20_C_60_, as well as the Met-Cars, Y_8_C_12_ and La_8_C_12_. | | | | |
| --- | --- | --- | --- | --- |
|  | Volleyballenes | |  |  |
| Models | Y_20_C_60_ | La_20_C_60_ |  |  |
| *E_b_* (eV) | 6.622 | 6.565 |  |  |
|  | Met-Cars | | | |
| Models | Y_8_C_12_ (*D*_2_*_d_*) | Y_8_C_12_ (*C*_3_*_v_*) | Y_8_C_12_ (*D*_3_*_d_*) | Y_8_C_12_ (*T_h_*) |
| *E_b_* (eV) | 5.999 | 5.987 | 6.046 | 5.499 |
| Models | La_8_C_12_ (*D*_2_*_d_*) | La_8_C_12_ (*C*_3_*_v_*) | La_8_C_12_ (*D*_3_*_d_*) | La_8_C_12_ (*T_h_*) |
| *E_b_* (eV) | 6.020 | 5.986 | 6.024 | 5.574 |

**Section II. *Ab* initio molecular dynamics**

|  | Potential Energy History | Dynamic Temperature |
| --- | --- | --- |
| **Y_20_C_60_** | 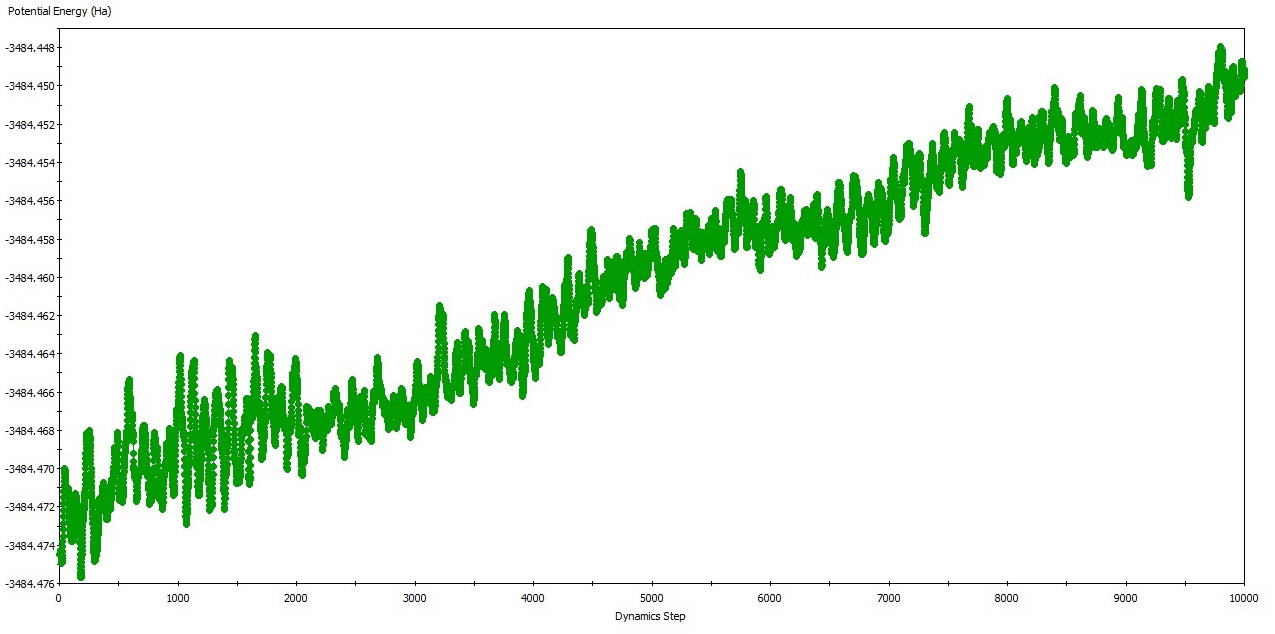 | 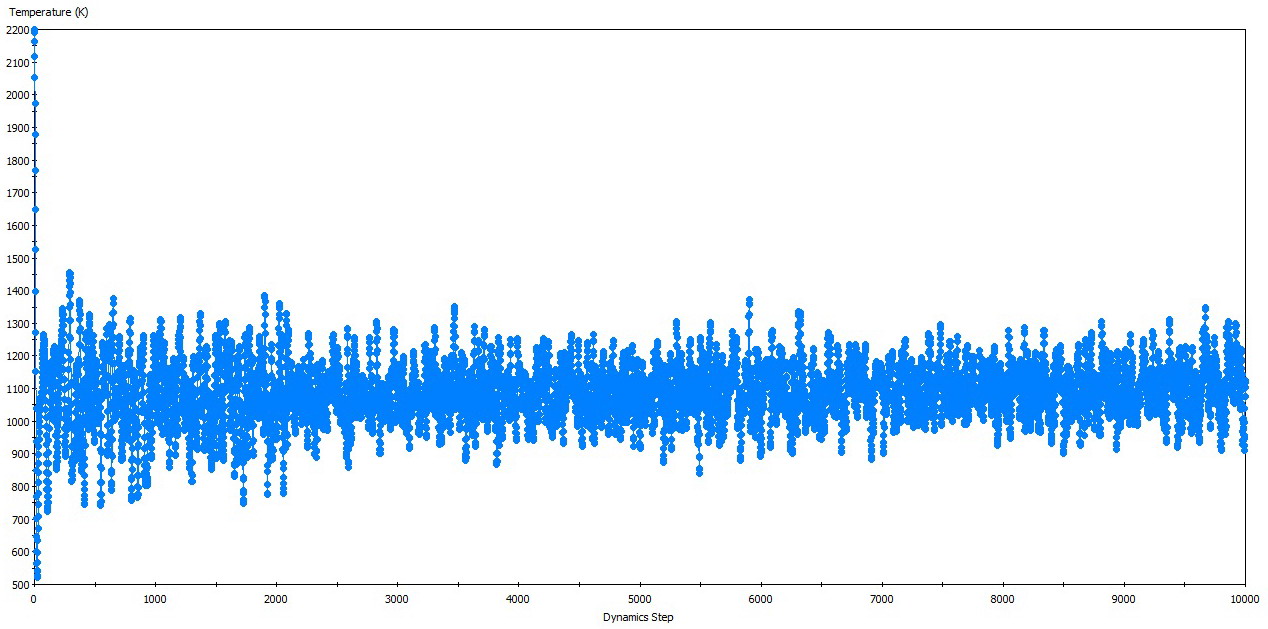 |
|  | NVE-2200K | |
| **La_20_C_60_** | 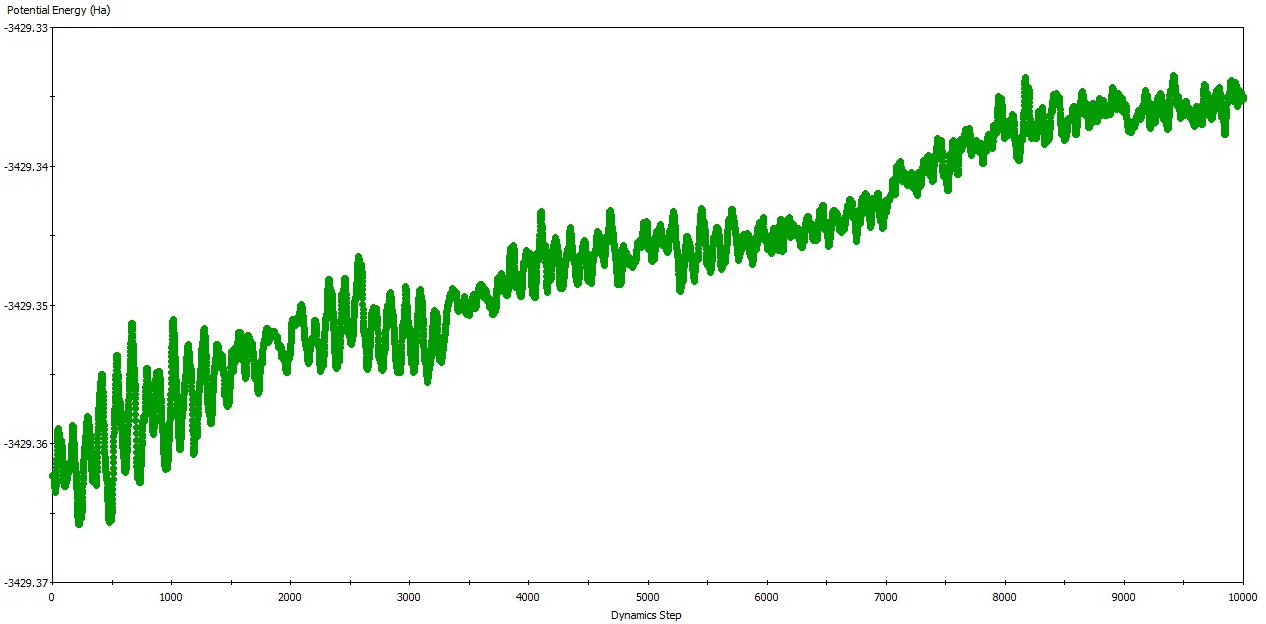 | 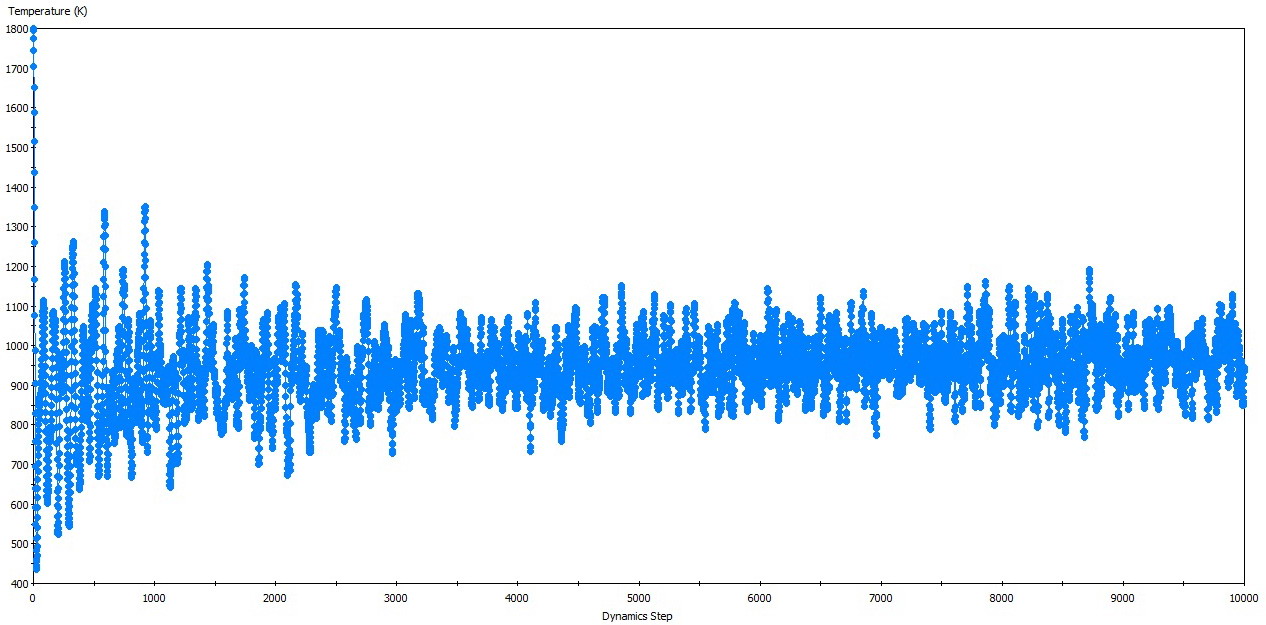 |
|  | NVE-1800K | |
| **Figure S1**. Results of *ab* *initio* molecular dynamics simulations with NVE ensembles at initial temperatures of 2200 K for Y_20_C_60_ and 1800 K for La_20_C_60_. The total simulation time was set to be 10.0 *p*s with a total of 10000 dynamics steps. The left two panels show the dynamic potential energy history and the right two panels give the temperature history *vs* the dynamic step. | | |

| 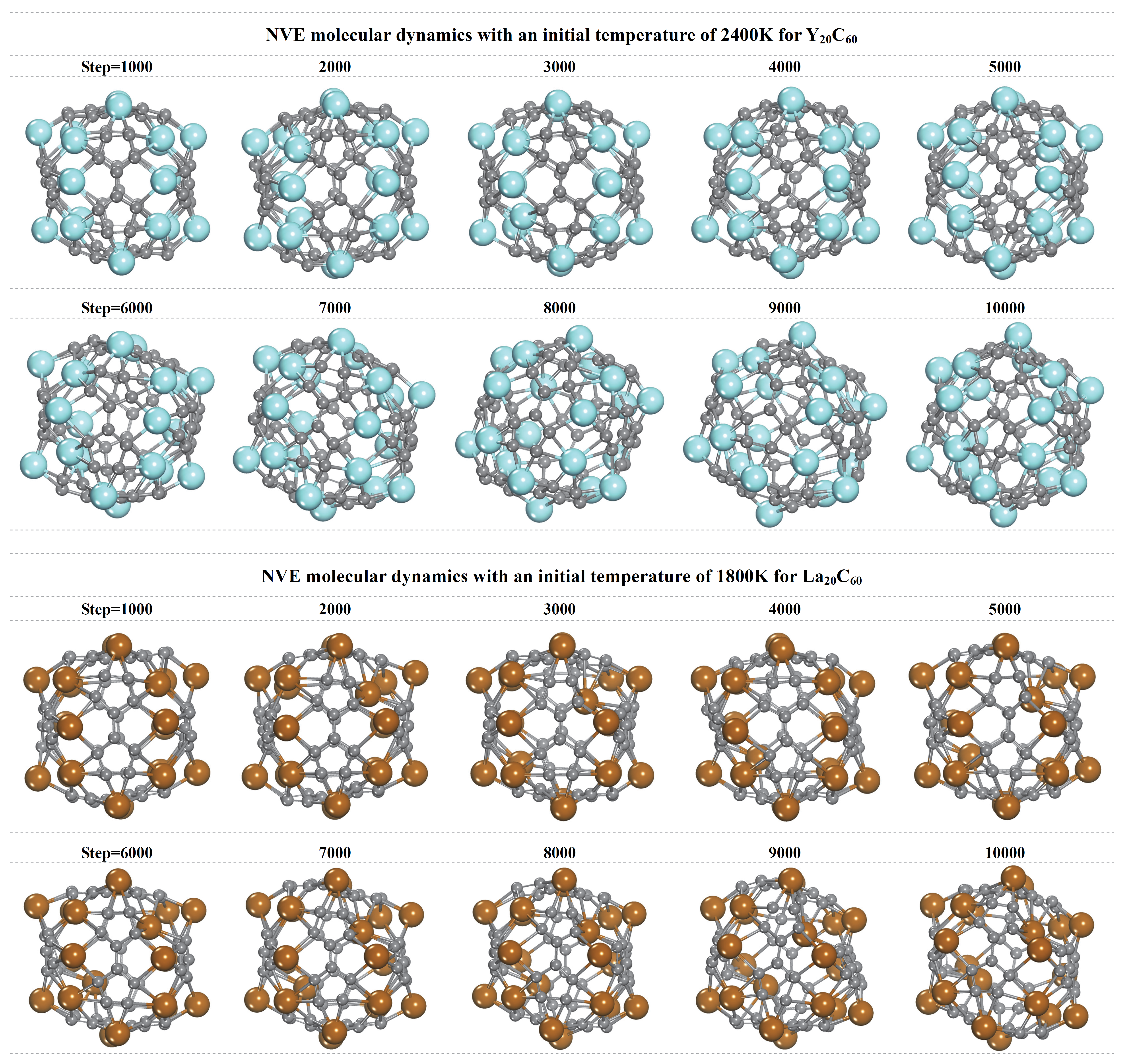 |
| --- |
| **Figure S2.** Selected frames at the 1000*th*, 2000*th*, 3000*th*, 4000*th*, 5000*th*, 6000*th*, 7000*th*, 8000*th*, 9000*th*, and 10000*th* step from the NVE molecular dynamics simulations with initial temperatures of 2200 K for Y_20_C_60_ and 1800 K for La_20_C_60_. |

**Section III.** **Some specific vibrational modes**

| **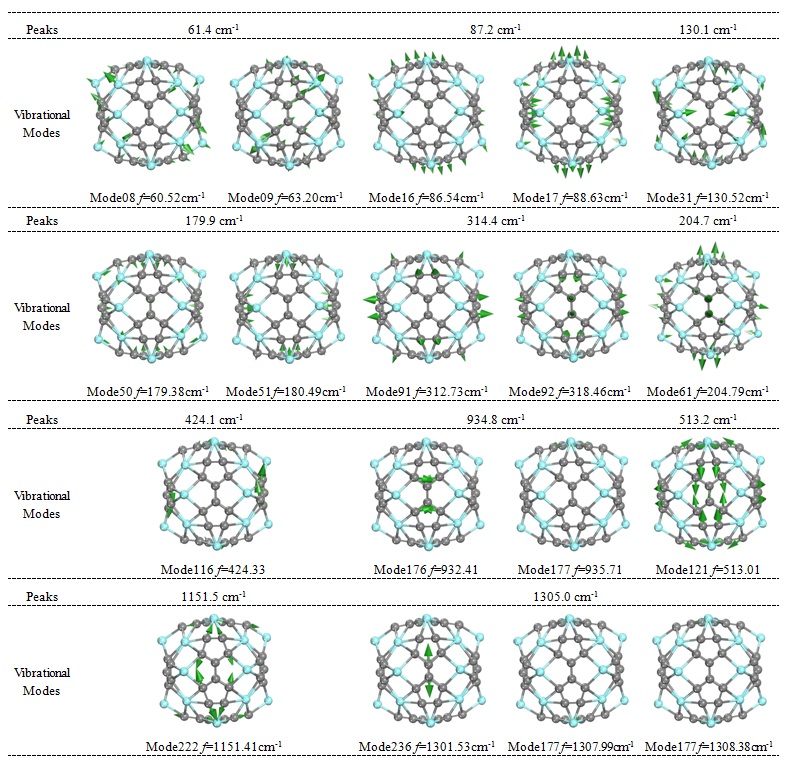** |
| --- |
| **Figure S3.** The specific vibrational modes corresponding to the peaks of the Raman spectrum for the Volleyballene Y_20_C_60_. Below each configuration the frequency is given. |

| **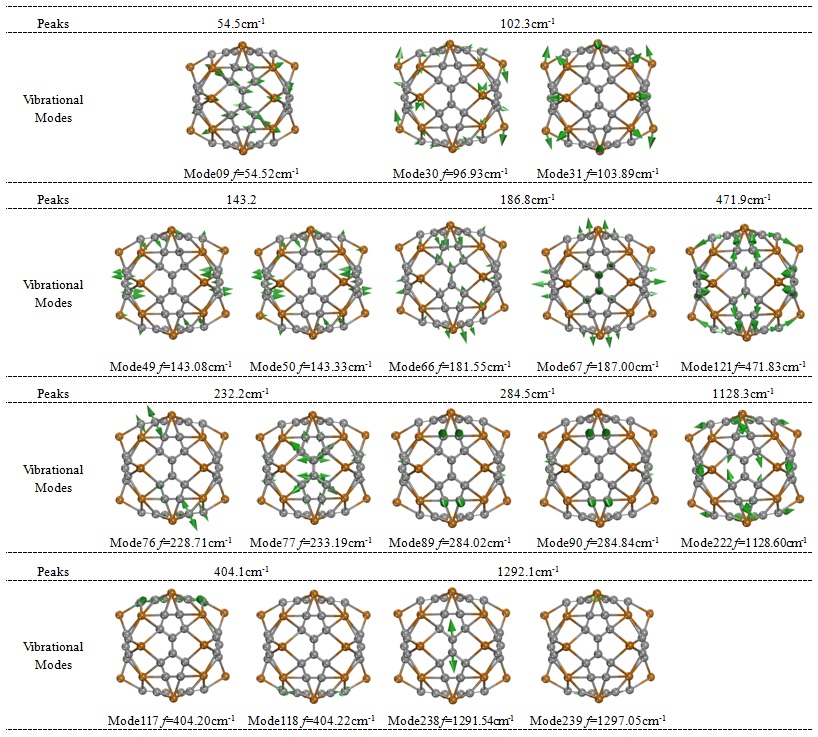** |
| --- |
| **Figure S4.** The specific vibrational modes corresponding to the peaks of Raman spectrum for the Volleyballene La_20_C_60_. Below each configuration the frequency is given. |

**Section IV. MD simulations in bulk**

In this section, we describe the *ab initio* molecular dynamics (MD) simulations with the constant-energy, constant-volume (NVE) ensemble that were imposed on the periodic Y_20_C_60_ and La_20_C_60_ systems. Here, the Volleyballene was placed in a cubic lattice with the values for the lattice parameter, *a*=*b*=*c*=15 for periodic Y_20_C_60_ system, and 16 Å for periodic La_20_C_60_ system. In addition, the dispersion correction for DFT, Grimme (Grimme, S. *J. Comput. Chem.* 2006, 27, 1787), was used in the MD simulation of the periodic Y_20_C_60_ system. The initial temperatures were set to 1800 K for Y_20_C_60_ and 1600 K for La_20_C_60_. The simulation time step was set to be 1.0 *fs* with a total of 5000 dynamics steps. The results of NVE simulations showed that over the course of a 5.0 *ps* total simulation, the topological structure was not disrupted. Figure S5 gives the results of two typical MD simulations, including schematic diagrams of the unit cell considered, the dynamic potential energy history *vs* the dynamic step, and the temperature history *vs* the dynamic step.

|  | **Periodic Y_20_C_60_** | **Periodic La_20_C_60_** |
| --- | --- | --- |
| **Unit Cell** | **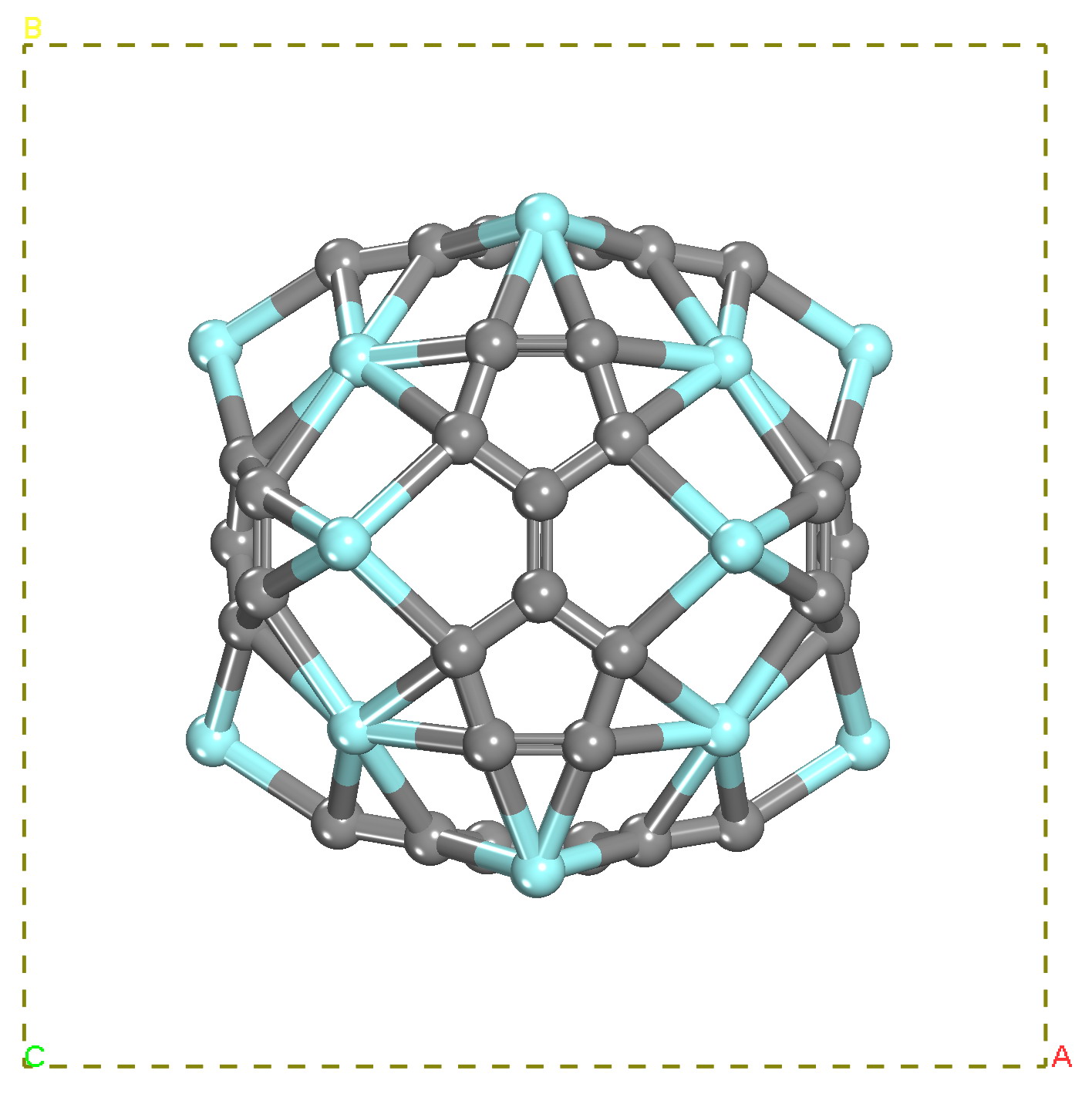** | 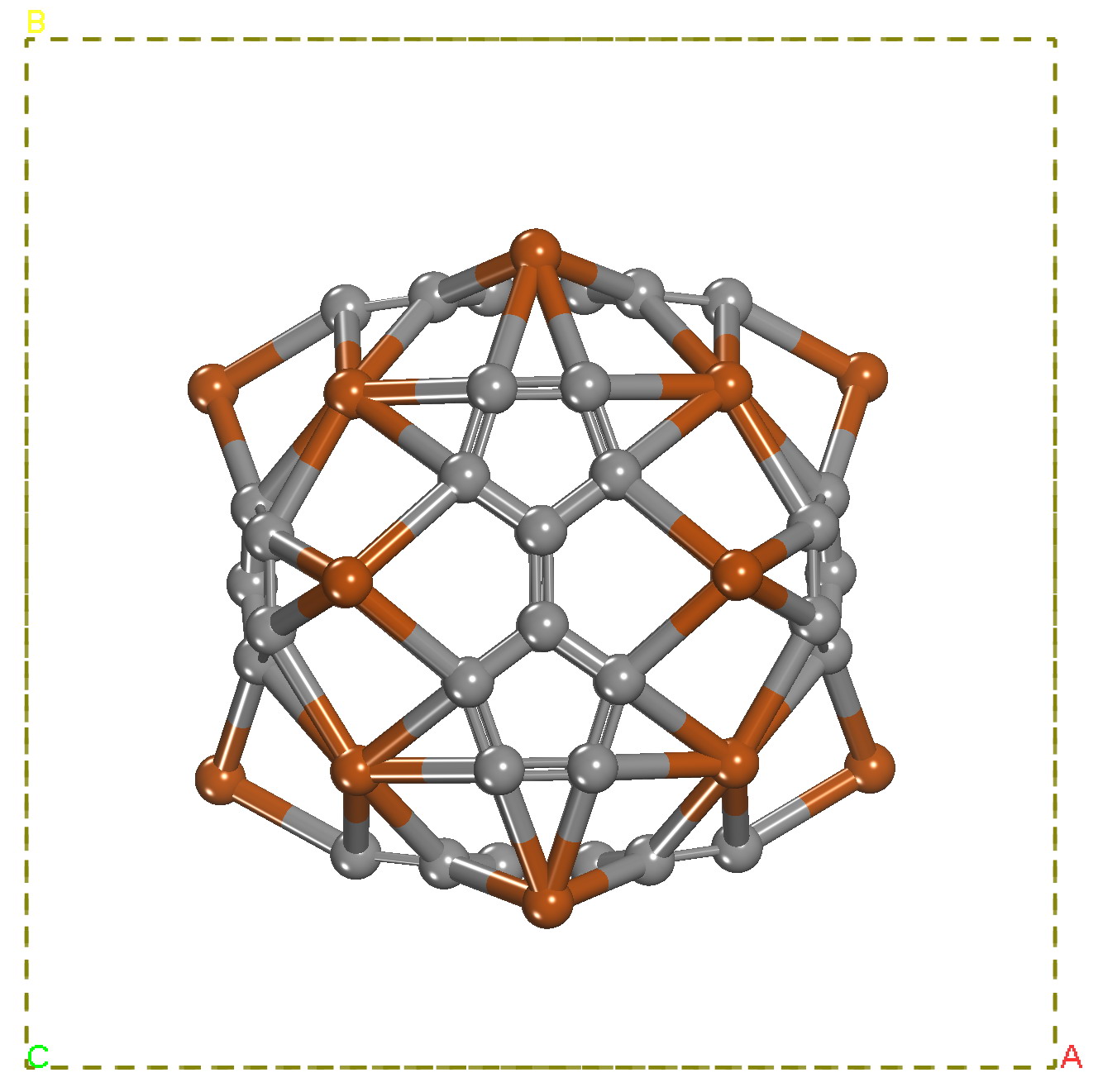 |
|  | ***a*=*b*=*c*=15Å** | ***a*=*b*=*c*=16Å** |
| **Potential Energy History** | 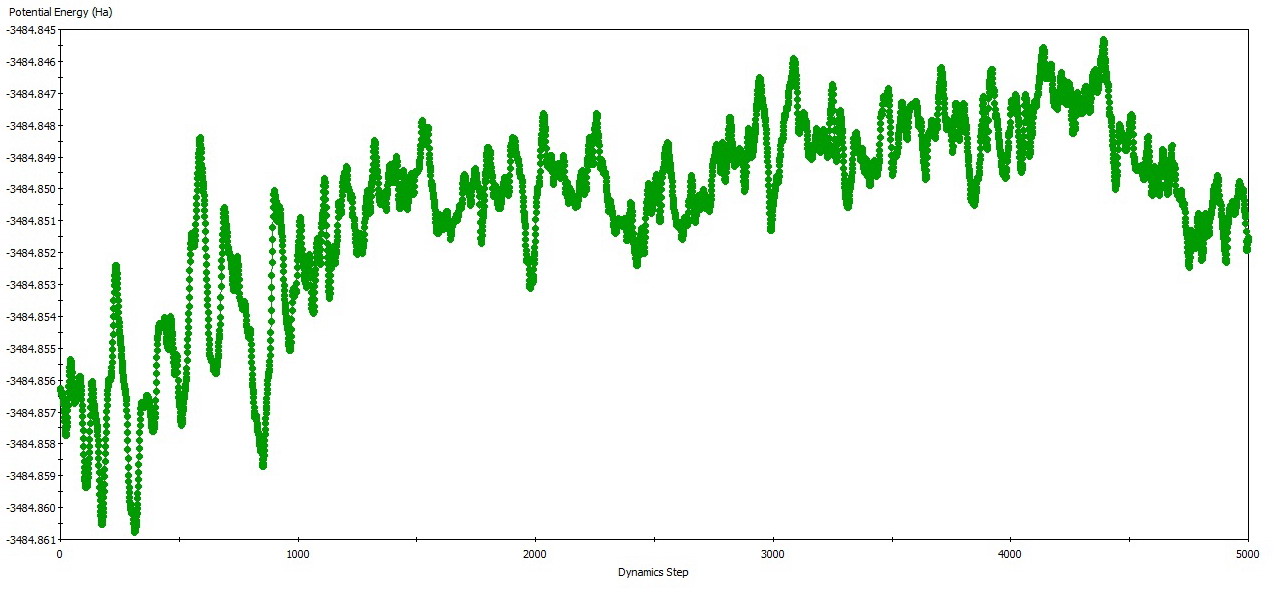 | 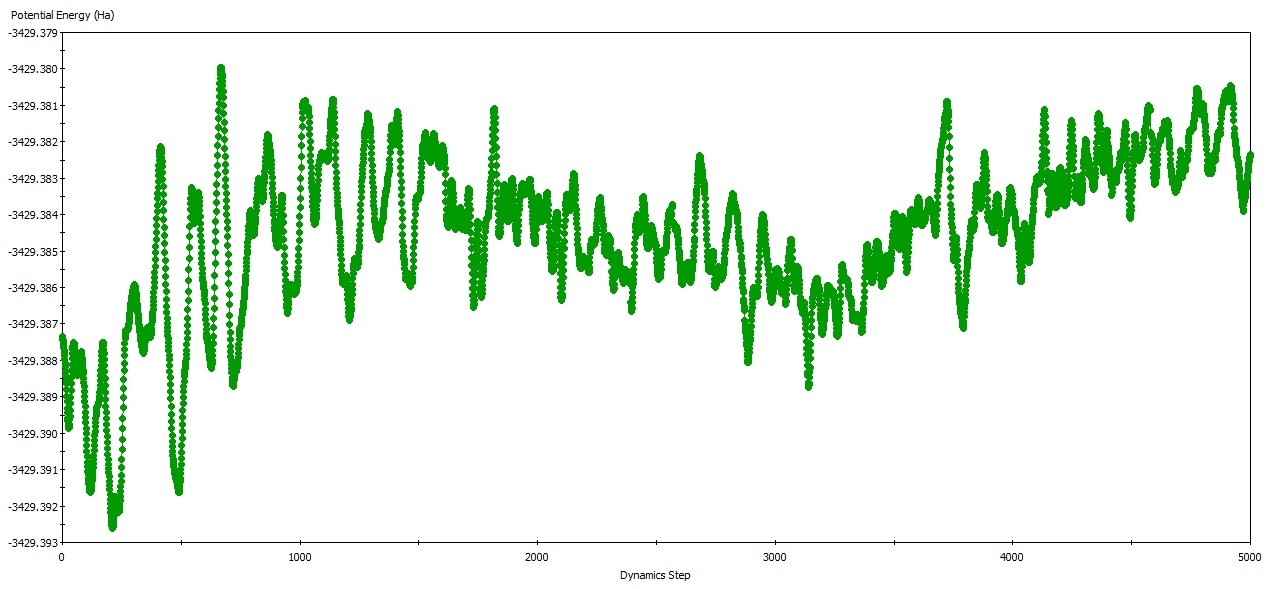 |
| **Dynamic Temperature** | 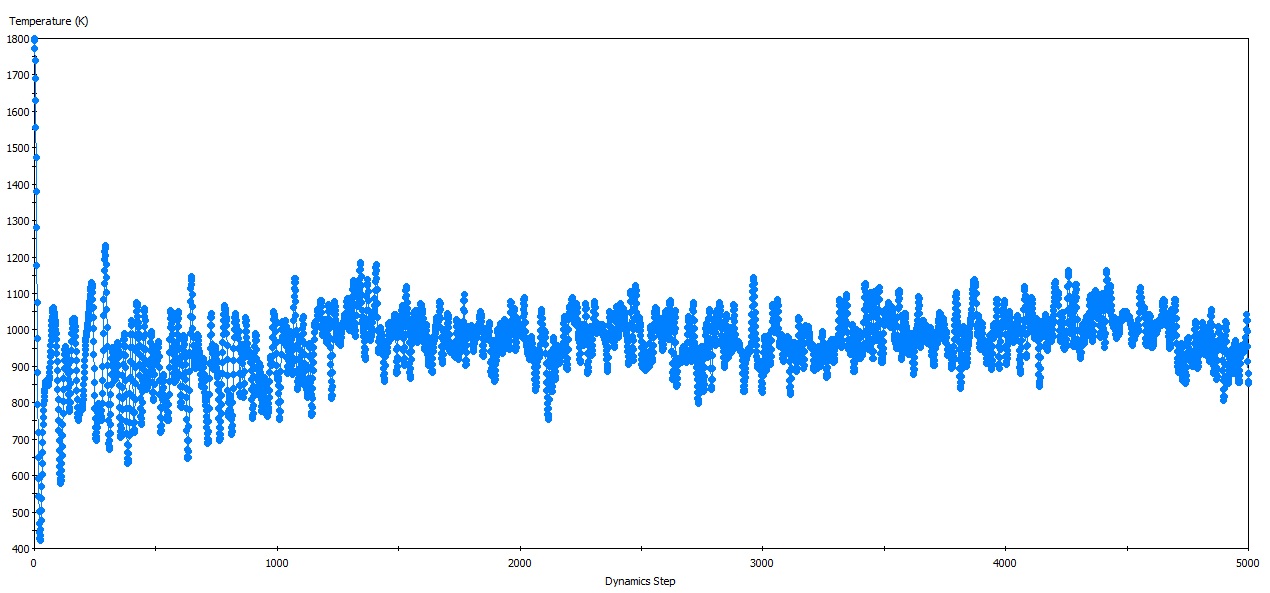 | 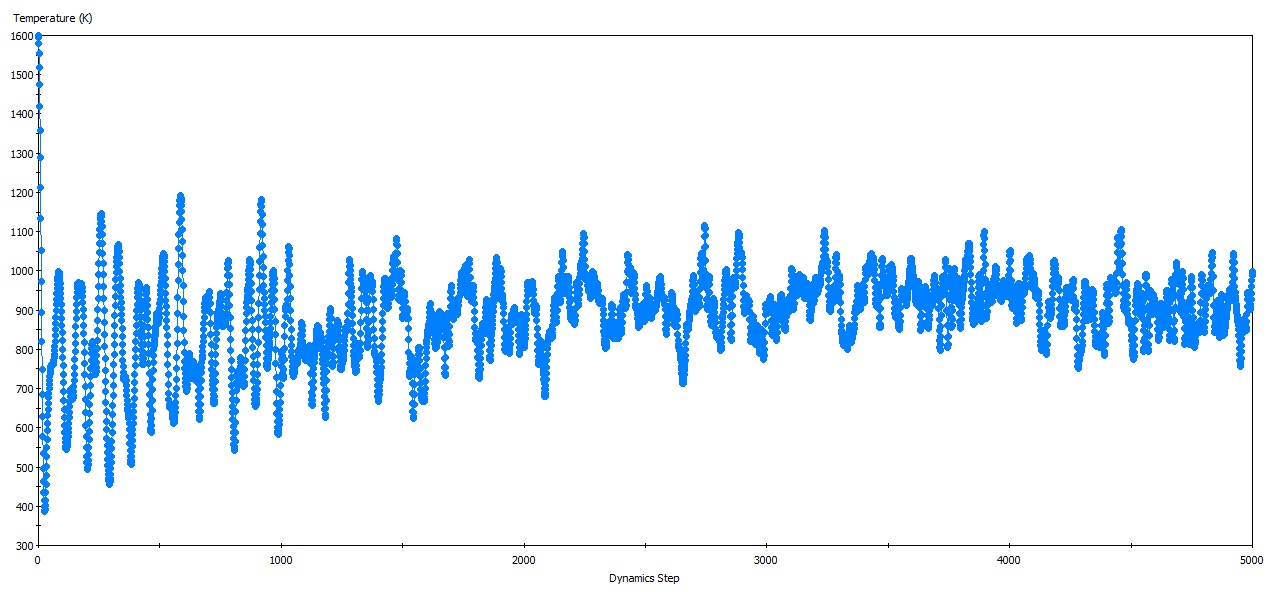 |
|  | NVE-1800K | NVE-1600K |
| **Figure S5**. Results of *ab* initio molecular dynamics simulations with NVE ensembles for the periodic Y_20_C_60_ and La_20_C_60_ systems. Included are the unit cell, the dynamic potential energy history *vs* the dynamic step, and the temperature history *vs* the dynamic step. | | |

In addition, the process of dimerization was considered for Y_20_C_60_+Y_20_C_60_ and La_20_C_60_+La_20_C_60_ binary systems. The Y_20_C_60_+Y_20_C_60_ or La_20_C_60_+La_20_C_60_ binary system calculations started with the selection of four sets of initial configurations which differed in the positions of molecules in the neighborhood of the point of closest approach. Figure S6 shows a schematic diagram of two typical portions of the Volleyballene molecule, the Sc_8_C_10_ subunit and the Sc_3_C_6_ slice. The four initial configurations were then defined as: (*a*) two Sc_8_C_10_ subunits directly facing each other with metal atoms facing toward metal atoms and carbon facing toward carbon; (*b*) two Sc_8_C_10_ subunits facing each other, but with the two Sc_8_C_10_ subunits rotated through 90 degrees with respect to each other, resulting in some metal atoms and carbon atom facing each other; (*c*) two Sc_3_C_6_ slices directly facing each other with metal atoms facing toward metal atoms and carbon facing toward carbon; (*d*) two Sc_3_C_6_ slices facing each other, but one Sc_3_C_6_ slice rotated through 60˚ with respect to the other, resulting in metal and carbon atoms facing each other, except for the two center metal atoms still facing each other.

| **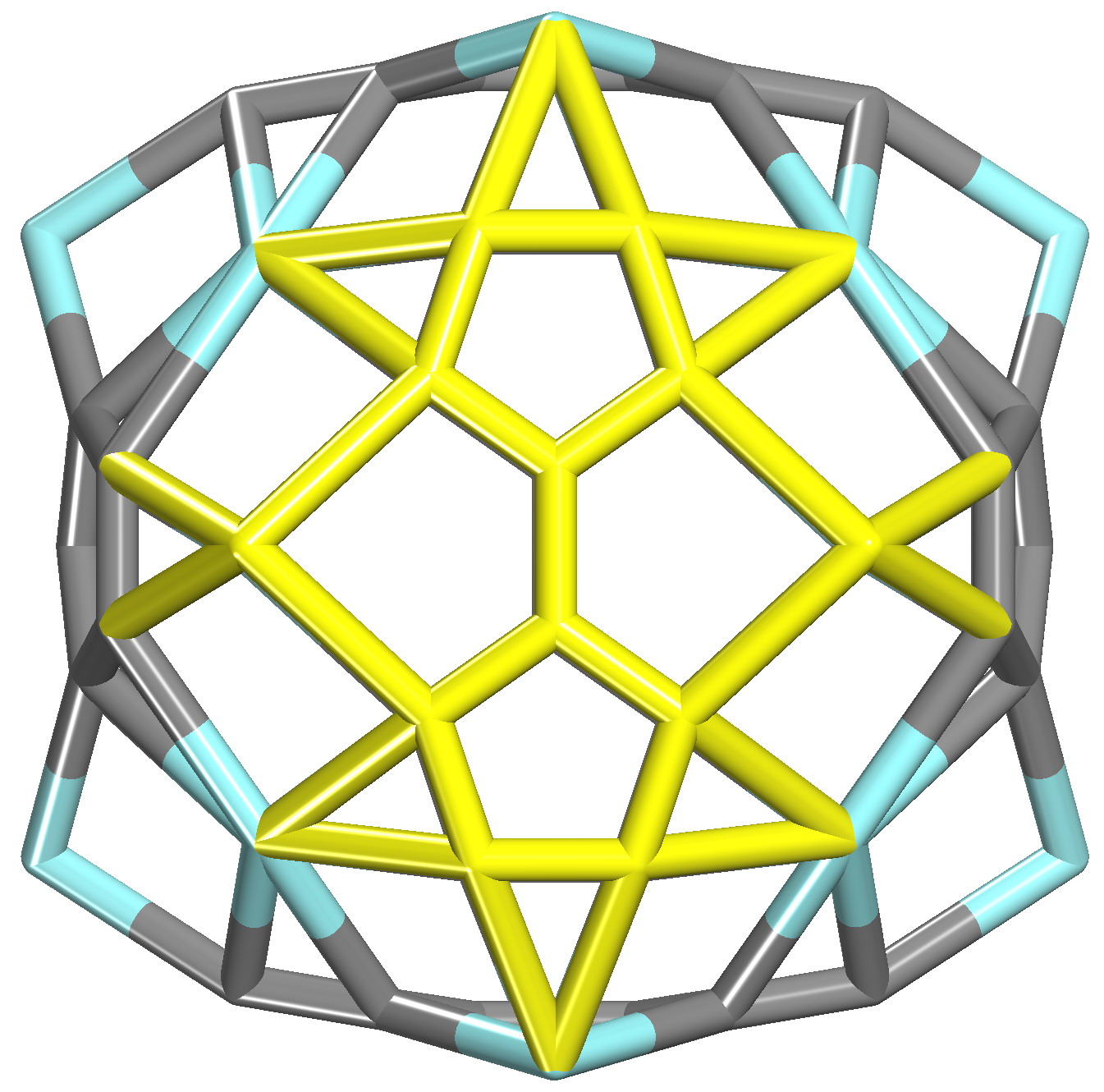** | **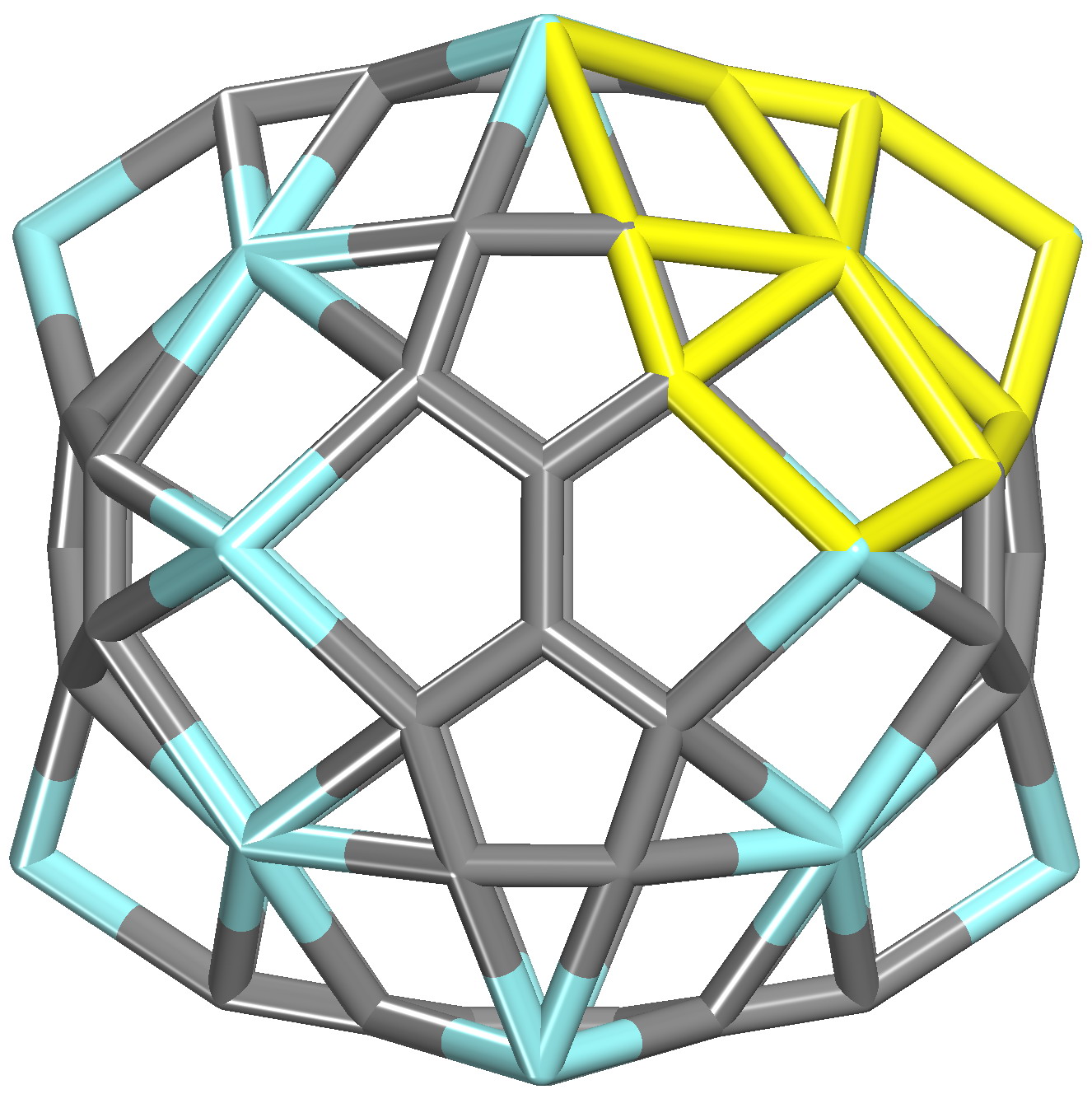** |
| --- | --- |
| (*a*) | (*b*) |

**Figure S6.** Schematic diagrams of the two typical parts of a Volleyballene molecule. The highlighted parts are the Sc_8_C_10_ subunit in panel (*a*) and the Sc_3_C_6_ slice in panel (*b*).

By calculating the binding energy of the binary system at each point with 15~20 different initial intermolecular distances as shown in Fig. S7, it was found that the binding energy per atom increased as the intermolecular distance decreased for cases (*a*) and (*c*), in which the metal atom faces another metal atom and carbon faces carbon. This indicates that *M*-*M* bonds between two Volleyballenes are difficult to form. It is worth noting that the binding energy per atom first decreases and then increases as the intermolecular distance increases for cases (*b*) and (*d*) as well, indicating that the two Volleyballenes do not easily aggregate. Typically, for case (*d*), there exists an equilibrium point at a particular intermolecular distance, *ie.* for metal to metal distances of 2.7 and 3.0 Å for the Y_20_C_60_ and La_20_C_60_ binary systems, respectively. The deformation electron densities were also calculated for the four typical Y_20_C_60_+Y_20_C_60_ and La_20_C_60_+La_20_C_60_ binary systems given above, *ie.* the structures for the equilibrium points in Fig. S7 (*b*) and (*d*), and listed in Fig. S8. From the deformation electron densities, it can be seen that there are no bonds between the intermolecular atoms, for either *M*-*M* or *M*-C.

|  |
| --- |
| **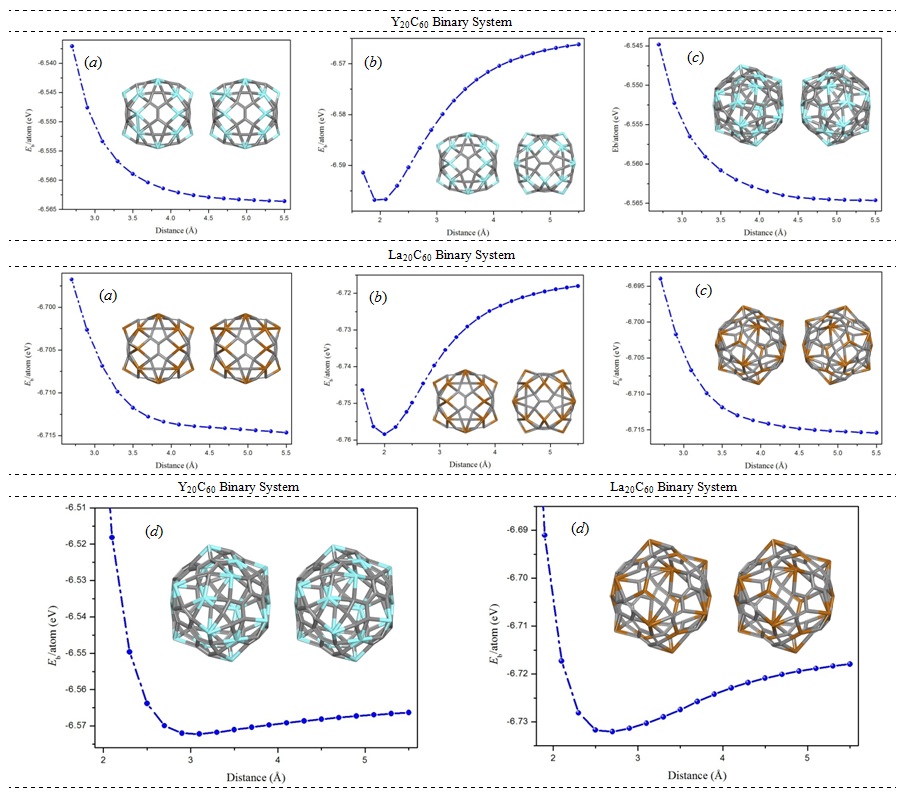** |
| **Figure S7.** Binding energy per atom (eV) *vs.* distance *d* (Å) of the Y_20_C_60_+Y_20_C_60_ and La_20_C_60_+La_20_C_60_ binary systems. The inset gives the initial configurations. |

| 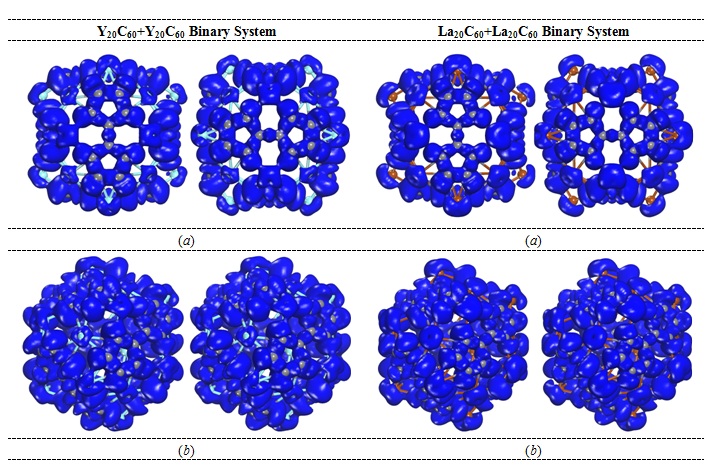 |
| --- |
| **Figure S8.** The deformation electron densities of four typical Y_20_C_60_+Y_20_C_60_ and La_20_C_60_+La_20_C_60_ binary systems. Parts (*a*) and (*b*) correspond to the structures at the equilibrium points in Figure S7 (*b*) and (*d*), respectively. The isosurface is taken to be 0.02 e/Å^3^. |
